# Recent Patents of Interest to Dentists

**Published:** 1906-09

**Authors:** 


### RECENT PATENTS OF INTEREST TO DENTISTS.

826,818, Dental tool, James H. Abbott, Philadelphia, Pa.
826,922, Tongue support, Elmer E. Davis, Los Angeles, Cal.
826,629, Cord-suspension electric dental engine, John V. Trenaman, New York.
826,977, Dental swaging device, George J. Weber, Liberty Center, Ohio.
827,507, Dental instrument, Lyter H. Crawford, Philadelphia,
827,508, Dental Swaging instrument, Lyter H. Crawford, Dalls, Texas.
827,236, Combined rubber dam clamp and holder, Harold J. Hansen, La Crosse, Wis.
827, 308, Tooth brush holder and sterilizer, David M. Hitch, Lasndowne, Pa.
827,693, Hypodermic syringe, Frederic W. Korb, Cleveland, Ohio.

827,121, Tooth-cleaning device, Patrick F. Roach, Westfield, Mass.

827,965, Tooth-brush, Gregor Engel, Charlottenburg, Germany.

828,109, Apparatus for stamping and shaping dental and metal crowns, Adolf Grumstein, Budapest, Austria-Hungary.

828,146, Forceps, James Somers, San Juan, Cal.

827,824, Dental articulator, Frederick W. Stephan, Chicago,

Copies of above tatents may be obtained for ten cents each, by addressing John A. Saul, Solicitor of Patents, Fendall, Building, Washington, D. C.

